# Qualifying deportation: How police translation of ‘dangerous foreign criminals’ led to expansive deportation practices in Spain

**DOI:** 10.1177/09670106221118798

**Published:** 2022-11-02

**Authors:** Barak Kalir

**Affiliations:** University of Amsterdam, the Netherlands

**Keywords:** Deportability, illegalized migrants, police, racism, Spain

## Abstract

In 2009, in a move to improve the situation regarding the deportability of illegalized migrants in Spain, a left-wing government led by the Socialist Workers’ Party drafted a new policy aimed at focusing police efforts exclusively on the deportation of ‘foreign criminals’. Ethnographically tracing the enforcement of deportation by a central police unit in Madrid, this article shows how the practical implementation of a policy that seemingly sought to limit the use of deportation in fact allowed for continuous and even reinvigorated deportation practices aimed at all categories of illegalized migrants. Operating under the idea that they were now fighting ‘dangerous criminals’, many police agents felt increasingly motivated about carrying out deportations and reassured about the morality of doing so. Rather than focusing on illegalized migrants who had been indicted for serious crimes, most police agents considered anyone with a police record to fit their target group. As a result of the specific police interpretation of the new policy, the deportability of illegalized migrants in Spain was not only increased but also left to be enforced according to the racialized and racist ideas of police agents. The article argues for the need to scrutinize all new deportation policies within Western liberal states in terms of their effect on deportability by highlighting entrenched and institutionalized forms of racism against illegalized migrants within the police force.

## Introduction

Deportation has been increasingly designated as the preferred policy instrument for dealing with unauthorized immigration or so-called failed or bogus asylum-seekers (Anderson et al., 2013; De Genova and Peutz, 2010; Weber, 2014). This is by no means a self-evident development. For starters, in spite of immense investment in its execution, deportation has proven to be a widely ineffective measure for solving the purported problem presented by irregular migration (Galvin, 2015; Hagan et al., 2008; Kalir, 2010). More importantly, as a legal sanction, deportation inflicts a hugely disproportional level of harm on people whose only violation of the law is administrative in character (Kanstroom, 2007; Menjívar and Abrego, 2012; Schuster, 2005). Despite committing no criminal act, illegalized migrants^1^ can be subjected to lengthy administrative detention, separation from their family, discontinuation of their work and/or education, and violent deportation, often to life-threatening destinations. The disastrous social, economic and emotional consequences of a possible deportation translate into a constant state of anxiety in the lives of millions of illegalized migrants, who must deal with the excruciating condition of ‘deportability’ (De Genova, 2002).

Next to sanctioning the deportation of illegalized migrants, the production of migrant deportability can serve various other goals for multiple actors. What deportability is and what deportability does for those who are involved in managing it, including migrants, can be two very different things (Franck and Vigneswaran, 2021). Moving beyond the treatment of deportability as a fixed structural condition, this article analyses the agency of actors who shape the social production and enforcement of deportability in practice.

The staggering disparity between an administrative violation and an immense sanction has led many Western states to adapt deportation policies in two principal directions. On the one hand, we witness a push towards the criminalization of migration and the expansion of the penal system to encompass administrative violations in the immigration field. This regressive trend, which Stumpf (2006) has termed ‘crimmigration’, is becoming globally popular (Bosworth et al., 2018) and has certainly been instituted to some degree in European countries like Spain (Fernández-Bessa and Brandáriz-García, 2016). On the other hand, there have been more progressive attempts to improve the situation regarding the deportability of illegalized migrants who are otherwise law-abiding and in the process of integrating into local communities and the labour market. This is done in some states by designing special schemes – such as those regulating DREAMers in the USA (Batalova and Mittelstadt, 2012) or *duldung* in Germany (Kirchhoff and Lorenz, 2018) – to partially tolerate and conditionally integrate certain types of illegalized migrants. More often, however, states deliberately turn a blind eye to the presence of ‘ordinary’ illegalized migrants, while using deportation more exclusively to target ‘foreign criminals’ (Fekete and Webber, 2009; Hasselberg, 2014).

While in some respects these policy adjustments can be seen as pushing in opposite directions – one towards increasing penalization, the other towards increasing tolerance – they both start out, on a deeper level, from a recognition of migration as a national security concern. Indeed, in recent decades, the ‘securitization of migration’ (Huysmans, 2000) has dominated policymaking in and around the issue of international border crossing and human mobility between countries (Bourbeau, 2011; Inda, 2006). According to this hegemonic doctrine, migrants and refugees are first and foremost perceived and treated as a potential risk to the security of the receiving nation-and-state. The risk can be multifold, as migrants can allegedly pose a threat to the cultural integrity of the nation, the stable composition of the labour market, the financial viability of the welfare system, the national security of the state and the personal safety of the latter’s citizens.

In a political climate that is increasingly anti-immigration and often straightforwardly xenophobic (Kalir, 2015), it is important to analyse the effects not only of more expansive deportation policies, but also of the few attempts at working in a more progressive fashion. In this article, I examine the move by the Spanish Ministry of the Interior to introduce to the deportation field a brand-new category: ‘qualified expulsions’ (*expulsiones cualificadas*). According to the ministry, the category of qualified expulsions pertained to the removal of ‘repeat offenders, perpetrators of crimes of serious violence or gravity, terrorists, prisoners whose punishment is converted to expulsion, or criminals under preventive custody or prison sentence’ (Ministry of the Interior, 2010). This new initiative in Spain – taken by a left-wing government led by the Socialist Workers’ Party (PSOE) – was intended to signal a systemic reorientation in the policing of deportation towards a much narrower target group. The police, purportedly, were no longer interested in illegalized migrants per se, but rather focused on deporting foreign delinquents who committed serious crimes.

In the years following the introduction of the new policy, qualified expulsions constituted, according to the Spanish authorities, the majority of all deportations executed by the police. Contrary to these official claims, however, this article will demonstrate how the use of qualified expulsions has in fact allowed for continuous and even reinvigorated deportation practices aimed at all categories of illegalized migrants. Accordingly, the anxiety that deportability generates in the lives of illegalized migrants was amplified by this alleged progressive policy move. To understand how a policy shift that seemingly set out to have a limiting role could contribute to the perpetuation and expansion of existing deportation practices, we need to pay attention to the ways in which police agents interpreted the new category of qualified expulsions on the ground.

Like many other state projects, deportation can suffer from an implementation deficit or surplus, depending on the availability of resources to execute particular policies, as well as the formal and informal practices of those who are charged with their implementation (Amit, 2020; Ellermann, 2008; Hall, 2012; Kalir, 2017a). The deportation field is particularly interesting in this respect because, as I stress elsewhere,

governments often deliberately delegate extensive power and discretion to the executive branch in order to pursue levels of implementation that are impossible to draft as formal regulations. This is because they are either in violation of international law and human rights conventions . . . or are plainly too racist and violent to be publicly announced. (Kalir, 2019a: 71)

The discretionary power of state actors is hardly ever employed in a random manner or simply according to the whims of individual bureaucrats. Examining the work of immigration officials in the USA, Heyman (1995: 263) argues that while ‘bureaucracies are hierarchical organizations designed to force the production of thoughts as a work duty . . . bureaucratic workers . . . must be controlled as thinkers’. Training programmes, organizational culture, peer learning, political pressure, religious views and many other social dynamics can all influence the ways in which discretionary power gets moulded and put into use in the implementation of formal rules and regulations (Kalir et al., 2012). Especially around sensitive issues, state functionaries do not always ‘think without a banister’, as Arendt (2018) would have them. Instead, they are more likely to act according to ethical ideas that are not, at least not entirely, of their own making (Waxman, 2009). Getting socialized into a particular modus operandi in policing is essential to the profession (Karpiak and Garriott, 2018), and in the immigration and deportation field it can be decisive in the application of discretionary power (Armenta, 2017; Cacho, 2012; Hasselberg, 2014; Kalir, 2019a).

Between 2016 and 2017, I conducted ethnographic research into the implementation of deportation policies in Spain. For a period of five months, together with a research assistant, I studied the daily work of police agents in the Brigade of Aliens and Borders (hereafter, BAB) in the province of Madrid. The BAB is the main police unit responsible for deporting illegalized migrants – mostly from the metropolitan area of Madrid, but also from other provinces throughout Spain.^2^ There are two subunits working at the BAB on the deportation of illegalized migrants. One subunit is responsible for processing illegalized migrants who are in pre-removal detention centers, but also sometimes takes part in operations aimed at arresting illegalized migrants in their homes (if the address is known to the police) or in public spaces. The other subunit focuses on illegalized migrants who are currently in prisons and should be targeted for deportation upon release. There are around 12–15 agents working in each subunit. For months, we were allowed to shadow agents and observed their practices. We held many informal talks with most agents as well as lengthy one-on-one interviews. In addition, we also attempted to conduct fieldwork at the Brigade for Expulsion of Foreign Delinquents (*Brigada de Expulsión de Delincuentes Extranjeros*, hereafter BEDEX). This brigade, inaugurated in 2009 and located close to Barajas international airport in Madrid, was in charge of coordinating the deportation of non-citizens who had served a prison sentence in Spain and either became deportable upon completion of their sentence or qualified for substituting a part of their sentence to be served in their country of origin. Our fieldwork at BEDEX was prematurely terminated by the head of the brigade after one week.^3^

Our study crucially shows that in the transition towards a focus on qualified expulsions in Spain, there was little monitoring of police implementation. Consequently, the police used the discretionary power invested in their office to implement the new policy according to their own convenience and in line with an entrenched racist anti-immigration attitude. In practice, police agents adopted a working definition of qualified expulsions according to which simply having a police record fitted squarely into the new category. This meant that the police deported thousands of illegalized migrants who had never been convicted of a single crime. To be sure, a police record can be registered on any occasion that the police suspect a person has committed or intended to commit a crime. Even if the police eventually choose not to pursue the case or the person is found not guilty, a police record of an incident might be kept in the police registry. Accordingly, as a result of the specific police interpretation and practical implementation of qualified expulsions, illegalized migrants’ deportability in Spain was not only increased but also more arbitrarily left to the racialized and racist ideas of police officers.

This article therefore argues that an apparent regulative attempt to align deportations more strictly with the fight against criminality can in fact produce the exact opposite result. Focusing on ‘dangerous delinquents’ not only motivated and reassured the police about their moral conduct in carrying out deportations, but also detrimentally compounded a stereotypical link between illegalized migrants and criminality in the public imaginary, while de facto reinforcing the deportation of non-criminal illegalized migrants. This, then, leads me to consider the more indirect ways in which the promulgation of crimmigration can bolster the overall governance of illegalized migrants as a security matter, even in countries like Spain where migrant illegality does not constitute a criminal act. The (missing) link in understanding how a supposedly progressive policy in practice produces adverse effects can be found in an ethnographic exploration of institutional racism. If endemic levels of racism and anti-immigration sentiments are left unchecked in the field, especially among the agencies and agents responsible for implementation, then it should come as no surprise that top-down policies are over-implemented or executed in a particularly stringent fashion in the level of everyday policing.

The article begins with a detailed overview of recent developments in the Spanish immigration and deportation field, followed by an ethnographic account of the distinctive ways in which police interpretation of qualified expulsions contributed to the perpetuation and expansion of deportation practices aimed at all types of illegalized migrants. I conclude by highlighting the role that institutional racism plays within police units in the Spanish immigration and deportation field.

## The Spanish context

Previously an emigration country for centuries, Spain has turned into a potent immigration destination since the early 1990s. Experiencing strong economic growth, not least in some heavily informal sectors like construction, agriculture and caregiving, Spain has attracted millions of unauthorized and authorized migrants, who have enjoyed a lenient visa and residency regime (Aja and Arango, 2006; Calavita, 2005). Like many other countries in Europe and beyond, Spain turned a blind eye to the entrance and permanence of unauthorized migrants, especially during periods of high demand for cheap labour (Martínez, 2004). In the mid-2000s, the number of illegalized migrants residing in Spain was guesstimated at around 1 million (González-Enríquez, 2009). After joining the European Union in 1986, Spain had to increasingly align with a move towards approaching migration as a security issue (Moffette, 2018: 95–99). Among other things, this meant that it began to fortify its physical borders (mostly in Ceuta and Melilla, the two Spanish colonial enclaves in Morocco), restrict work and residency permits, and actively illegalize unauthorized migrants (Calavita and Suarez-Navaz, 2003).

With respect to deportation, since the early 2000s Spain has consistently been among the top EU states in issuing deportation orders to non-EU citizens and executing a high number of ‘enforced returns’ (*Eurostat*, 2018). Although the number of deportees has fluctuated, since 2000 Spain has carried out between 10,000 and 15,000 deportations each year. Since 2013, the number of deportees has been decreasing, mostly as a result of an economic slowdown that reduced the number of newcomers and pushed some long-term migrants to independently leave the country or accept so-called voluntary return programmes (Kalir, 2017b). Thus, in 2013, the total number of deportations in Spain stood at 8984, in 2014 at 7696, in 2015 at 7696, and in 2016 at 5051 (Ministry of the Interior, 2014, 2015, 2016).

Deportations constitute a politically heated and publicly sensitive topic in Spain. The Spanish media have frequently reported on incidents concerning police mistreatment of illegalized migrants, mostly during street arrests, in detention centres and on deportation flights. In recent years, a number of migrants have died inside detention centres, leading to formal investigations by lawyers and the Ombudsman, as well as public protests regarding police violence.^4^ Organized campaigns were initiated in Spain by different activists and civil society actors, calling for the closing down of all detention centres and for the abolition of deportation flights (see, for example, Campaña Estatal por el Cierre de los CIEs, 2014; Observatorio Criminológico del Sistema Penal ante la Inmigración, 2017).^5^ Another contentious point has been the border crossings in Ceuta and Melilla. With strong financial and ideological support from the EU Commission, Spain invested heavily in fortifying its borders with Morocco to prevent migrants and refugees from crossing into its sovereign territory (López-Sala, 2015). With the rise in the number of refugees from the Global South arriving in Europe, deportation of so-called failed or bogus asylum-seekers has been insistently construed as crucial for ensuring the safety of the native population and attending to the case of so-called real refugees.

In Spain, as in many other Western states, ‘acting tough’ on immigration has been considered essential in preventing a ‘pull effect’ (*efecto llamada*) – that is, the alleged disposition of millions of migrants and refugees around the world to flood weak states. As part of its attempt to consolidate its tough stance against unauthorized immigration, Spain has regularly practised ‘hot returns’ (*devoluciones en caliente*) of people who crossed the borders into Ceuta and Melilla. In violation of international law, the Spanish Civil Guard (*Guardia Civil*), which is the police agency in charge of, among other things, guarding territorial borders, deported back to Morocco people who managed to cross into Spanish sovereign territory. According to the law, these people had to be given an opportunity to file their asylum applications with the Spanish authorities. The illegal practice by the Spanish authorities was brought before the European Court of Human Rights in Strasbourg, which reprimanded the Spanish state and demanded the immediate termination of the activity.^6^ Nevertheless, ‘hot returns’ continue to be carried out by the Spanish authorities, who insist that Spain is within its sovereign right to reject those who are caught trespassing its borders illegally.

The uncompromising effort by the Spanish authorities to prevent migrants and refugees from reaching Spain’s sovereign territory has led to some deadly incidents. For example, in February 2014, at the beach of Tarajal in Ceuta, officers of the Spanish Border Guard shot rubber bullets and tear gas at a group of unarmed people who tried swimming over from the Moroccan side into the Spanish enclave. At least 14 people died in the incident, and dozens more were seriously injured. The case was brought to court in 2014, with Border Guard officials involved being charged with the murder of unarmed people at sea. In 2015, the case was dismissed by the ruling judge. The dismissal was later appealed by a few NGOs, and the case was reopened in 2017. Finally, in 2018, after years of legal procedures, the court in Ceuta decided to archive the case, finding no one guilty for what had happened in Tarajal.^7^

In analysing such deadly incidents, as well as more routine procedures that are part of a state’s efforts to act tough on immigration, we must always consider the position of street-level bureaucrats (Lipsky, 1980) who are charged with the implementation of legally and publicly disputed state policies (Bauman, 1991). Recent studies have highlighted the moral struggles of different frontline state actors who interact with deportable non-citizens in extremely challenging conditions (Fassin, 2005; Hall, 2012; Kalir, 2019a; Ticktin, 2006). State actors were often torn between their commitment to implement strict exclusionary state policies and the compassion they felt towards migrants and refugees in dire humanitarian circumstances. The police represents an interesting case in point here as its enforcement of the migration policies of local and national authorities often constitutes a ‘grey zone’ with a wide margin for discretionary power (Feldman, 2016, 2019).

The introduction of qualified expulsions to the Spanish deportation field presented police agents with a new type of moral struggle. Focusing exclusively on the deportation of dangerous criminals was clearly a more legitimate and worthy task for the police. The dilemma, however, was how to go about refraining from the deportation of illegalized migrants who were non-criminal but nevertheless suspected by the police of being ‘bad’ (*malos*). Policymaking and policing in the Spanish immigration field have often come under fire for reinforcing rather than diffusing existing institutional racism against migrants and refugees, especially those from Africa and Latin America (Arenas García and García España, 2016; Jueces para la Democracia, 2011). Swiftly aligning their policies with the global tendency towards the securitization of migration in Western states, Spanish authorities employed and hardened a negative and racialized image of the illegalized migrant, who came to symbolize the abject Other in society (Nyers, 2003).

Spain also plays an important role in the securitization of migration at the level of the European Union. In charge of controlling the influx of unauthorized migrants from Africa to Ceuta, Melilla and the Canary Islands, since the late 1990s Spain has received substantial support from the European Commission for this purpose. EU budgets, technological support and direct collaborations with Frontex have been employed to assist Spain to fence its land borders, patrol its maritime zones, and intensify the repatriation and deportation of illegalized migrants (López-Sala and Godenau, 2016). There is clearly an agreement between Spain and the EU on the need to invest in an ever more robust Spanish border and deportation regime. For example, in November 2014, Spanish Minister of the Interior Jorge Fernández Díaz met in Paris with Dimitris Avramopoulos, the newly appointed European commissioner for migration and home affairs. The two men discussed the continued support of the European Commission, and Frontex in particular, to Spain in its fight against unauthorized migration at its external borders. Fernández Díaz informed the new commissioner that ‘in the avalanche of irregular migrants entering Spain there are terrorists and jihadists’, while the commissioner reiterated the importance of Spain for the integrity of the EU borders (*elDiario.es*, 2014). Coming from the highest political echelons in Spain and the EU, this type of discourse and commitment to protecting Europe from ‘infiltrators’ undoubtedly contributed to the harsh enforcement of policies by agents on the ground.

## From ‘undocumented migrants’ to ‘dangerous criminals’?

At the level of Spanish politicians and policymakers, two obvious motivations underlined the move towards qualified expulsions and the refashioning of deportation as a fight against ‘dangerous criminals’. First, the government was clearly responding to intensifying public pressure to stop or drastically modify existing detention and deportation practices. Scores of media items and NGO reports had criticized the police for deporting illegalized migrants who were living in Spain, working hard, raising a family, and were socially integrated in their local communities. These reports portrayed deportation as a violent and inhumane police intervention that often separated families, causing trauma to young children and having severe negative repercussions for tens of thousands of ordinary people whose only ‘crime’ was an irregular civic status in the country.^8^

On another level, the introduction of qualified expulsions had a clear internal objective aimed at beefing up the motivation and morale of police agents in the field. It was no secret that many police agents considered immigration to be far removed from proper police work. In interviews I conducted with police agents at the BAB, it transpired that many of them ended up in the immigration field either because they were ‘green’ in the service and had no say about where they were stationed or precisely because they had been working very intensively for many years in other police units and were now seeking a more ‘boring’ position to wind down. Police agents mostly saw immigration as a field to which their professional skills were not best suited. Recasting police work on immigration as a fight against terrorists and serious foreign criminals was importantly meant to inject the field with a proper policing mission.

In 2010, after the first year in which BEDEX became operational, a report prepared by the Ministry of the Interior announced that in 2009 Spain had deported 7591 foreigners. The report further informed the public that 57% of all deportations were considered qualified expulsions, and that many of the deportees were serial criminals as they had committed between them a total of 23,918 crimes. Of these, 46% were property offences, 19% involved violence against people, 8% were offences against public order, and 5% involved gender-based violence. The police praised BEDEX for carrying out qualified expulsions in cases of ‘foreign delinquents with numerous criminal and judicial records, linked to terrorism, organized gangs, gender violence or any other type of serious crime’ (Ministry of the Interior, 2010).

**Figure 1.**
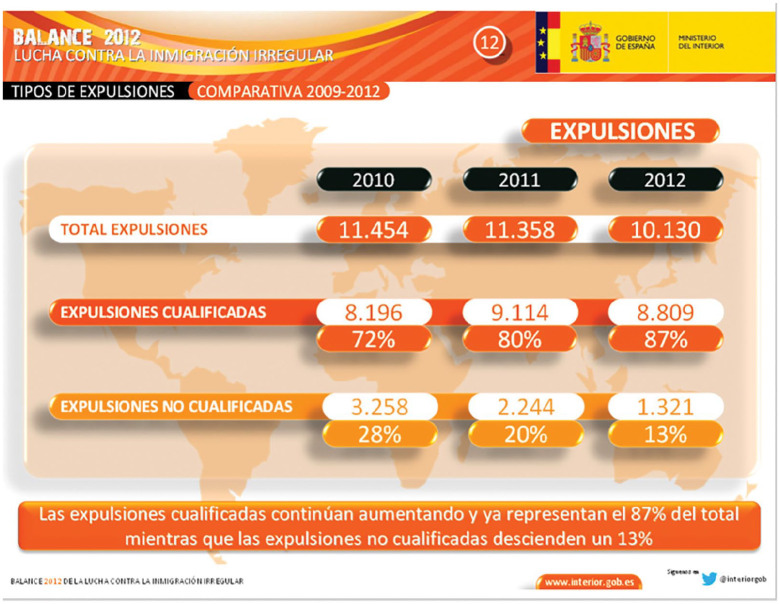
Types of expulsions (comparison 2009–2012). *Source*: Ministry of the Interior (2013).

In the following years, the ministry produced an annual report entitled ‘The Fight Against Irregular Immigration’, which included a table specifying the number of qualified expulsions and their proportion out of the total number of deportations that year (see Figure 1).

According to Figure 1, in the period 2010–2012, the number of total deportations per year decreased slightly, while the proportion of qualified expulsions went up each year, reaching a record high of 87% in 2012. In the years 2013–2015, the proportion of qualified expulsions fluctuated slightly but reportedly maintained a level of above 80% of all annual deportations (Ministry of the Interior, 2016).

The media in Spain picked up on the ministry’s annual reports. Qualified expulsions began to play a major role in the public framing of the debate around deportation, as newspaper articles and televised reports drew ever more sensationalist links between qualified expulsions and the presence of foreign criminals and terrorists in the country. These links have become visibly racialized, as many of the photos chosen to accompany news items regularly show black and brown people as alleged criminals being escorted by heavily weaponized police agents, the latter often wearing balaclava masks. Exemplary here is how in 2010 a few newspapers reporting on BEDEX operations chose to show a specific photo of two brown-skinned women arrested by the police, with a caption that read: ‘Network of prostitutes cracked by the National Police in Barcelona’.^9^ Importantly, prostitution was not a type of crime that BEDEX considered a priority in its implementation of qualified expulsions. Moreover, women in general and sex workers in particular comprised an insignificant percentage of the total number of detained foreigners and qualified expulsions (Gonzales-Beilfuss et al., 2018).

In 2016, I conducted an interview with the director of BEDEX, a woman in her mid-forties. The commitment with which BEDEX carried out its mission was unmistakable. The director was keen on sharing all sorts of statistics that demonstrated the achievements of the unit in deporting criminals, yet she also cautioned that dangerous foreign delinquents constituted only a small proportion of the entire migrant population in Spain. This nuance, however, was nowhere to be found in an interview with a senior BEDEX officer in charge of operations:

This work is essential and necessary. And when the time comes to reinforce this unit, it will have to be provided with more resources and more personnel, because it is necessary for the future of this country. Since the EU is not taking measures in relation to immigration, with terrorists and jihadists, I think things will get worse with time. . . . There are many immigrants from Arab countries, Muslims. . . . These are very dangerous countries, and, errmm, I don’t know how we’ll end up. I hope not to find out. I believe I won’t find out, but I’m saying this for [the sake of] our children and grandchildren, you get me? God knows what they will have to endure. (Interview 1)

It was difficult to verify whether BEDEX only deported people who had committed serious crimes, as the category of qualified expulsions stipulated. Yet, since the unit exclusively dealt with non-citizens who had served a prison sentence, it was certain that individuals deported by BEDEX would have a criminal or judicial record. The same, however, cannot be ascertained in the case of other police units in Spain, such as the BAB, to whose processing of qualified expulsions we now turn.

## From ‘criminal records’ to ‘police records’

Time moved slowly within the BAB. The pace of office work was unhurried, and agents moved around leisurely between offices, talking a lot to each other about both professional and non-professional matters. It was common to have a radio or even a TV set on throughout the day. Going together for a late breakfast around 11a.m. (a ‘second breakfast’) was a regular practice among some agents, as was the lunch break in which agents often went by car to a restaurant in a nearby suburb. The atmosphere among the agents and between them and their superiors in the unit was very friendly. Hierarchy was rarely tangible. Everyone seemed to know their work well, and any outstanding issues were mostly discussed by way of consultation.

Agents at the BAB expressed the view that work in the immigration field was ‘very little police-like’, as one young agent put it (Interview 2). Working at the BAB or most of the other units in the immigration field mainly involved deskwork: computer research, filing papers, making phone calls and sending emails. Nevertheless, agents seemed to be highly dedicated to their work. As one senior agent explained:

I was always more oriented towards operations. Before I was at drugs [operations] and on the street. This work in immigration is more bureaucratic. But I think that all police work is important, so whether you catch drug traffickers or deport delinquents, it’s all necessary for the country. (Interview 3)

A few BAB agents even went the extra mile in trying to incorporate more detective work into their job. In one BAB subunit, two agents were especially known for following illegalized migrants with ‘bad records’ on the streets of Madrid in order to secure their arrest at an opportune time for deportation. In this context, ‘bad records’ meant that illegalized migrants had acquired police and/or criminal records during their period of residence in Spain. This kind of operation was not part of the official mission of the BAB. When I asked one of the energetic agents about this proactive attitude, he told me that it was simply in line with his understanding of the role of a police agent in Spain.

In line with their overall commitment to the job, agents unequivocally praised the introduction of qualified expulsions to the field. When I asked them about it, agents made it clear that a focus on criminals infused their work with a more concrete sense of policing. Below are some examples of their comments:

The commander doesn’t have to tell us that we should focus on qualified [expulsions]. We know what our work is about. (Interview 4)

At a police level, it’s clearly more important to carry out qualified expulsions. (Interview 5)

In principle, a deportation is a deportation, but on a personal level it gives me much more satisfaction to deport a serial delinquent than someone who just had one case of theft five years ago or no charges at all. (Interview 6)

The police focus on qualified expulsions was evidently animated by a sense of a mission to fight criminality in Spain. It was also, however, significantly propelled by more pragmatic reasons. Targeting foreigners who had completed a sentence in prison or whose whereabouts were known to the police meant that the police could prepare for their deportation and arrest them at the most opportune time and place (see Brandariz-García and Fernández-Bessa, 2017).

Yet, as I observed the work in the BAB, it quickly became obvious that agents were deporting many illegalized migrants who did not fit the category of qualified expulsions. Occasionally, there were cases in which illegalized migrants who had no police or criminal records were deported. When asked about one concrete deportation that had just been carried out, a commander of one subunit explained:

Of course we deport also not qualified. Let’s say that someone lives here for ten years and he couldn’t legalize his status. So we have to deport him. I mean, ten years and you were not capable of legalizing yourself in the country?! Sorry, this is not my fault, so evidently you need to be deported. (Interview 7)

More importantly, it appeared that the majority of BAB agents systematically employed a different interpretation of what constituted qualified expulsions in the first place. Agents regarded having a police record as being enough to qualify an illegalized migrant as a ‘dangerous criminal’. Here is an example of how one agent explained his understanding and working definition of qualified expulsions:

Qualified is simply an expulsion of a criminal. Pepito [derogatory term for a migrant] has been detained by the police 28 times – for robbery, theft, sexual violence or who knows what. For the police, for public security and for the government, the idea is that this person should not be in Spain. Qualified is a person we don’t want here in Spain. Someone who has been arrested four times for sexual violence but never went to prison, because the wife decided not to make a formal complaint, because whatever. But these police records exist! And this person is not an angel, but – excuse my language – he is an asshole. (Interview 8)

As mentioned earlier, a police record can be filed for anyone who is stopped by the police on suspicion of having committed or planning to commit a crime. This means that even when the police decide not to pursue charges or fail to prove them in court, a police record might be kept in the police registry. In practice, then, the BAB implementation of qualified expulsions became largely self-referential. That is, if the police suspected and stopped a person on whatever grounds and made a record of it, that person could from that point onwards be classified by the police as having fulfilled the criteria for a qualified expulsion. It also means that if an undocumented migrant failed to provide his or her documents to the police during a check, the incident could translate into a police record that could then in itself constitute the ground for classifying that person as suitable for a qualified expulsion the next time he or she is stopped.

It is important to note here that the inclusion of police records in the practical understanding of what constituted qualified expulsions received considerable support from the Spanish state. As Martínez Escamilla (2016) has highlighted, in 2011 then acting Minister of the Interior Fernández Díaz openly advanced the idea that the policy of qualified expulsions applied to those who ‘had police or judicial records or both’. We should not underestimate how much the political clout of an acting minister may have influenced the police’s implementation of qualified expulsions on the ground.

Realizing the notable divergence between the formal policy regulating qualified expulsions and its actual implementation by the police, I asked the commanders of the two BAB subunits whether their work with respect to the number and proportion of qualified expulsions was subject to monitoring by higher-up instances. One commander immediately waved off my question, saying offhandedly, ‘I know how to do my job. If there are any issues with it, I’ll get to hear about it at the end of the year, but I can tell you that that never happens’ (Interview 9). The other commander also seemed nonchalant about the issue but gave a more elaborated response:

In principle, yes, I have to discuss our results with my commander every year. We make a statistical report every month. And, in general, we strive to have as many qualified as possible. But this does not only depend on us, so if we happen to deport more non-qualified in one month, that’s not a problem. I sometimes look at the statistics to see how we are doing. We don’t work with a quota, but we know more or less our average number per month. If we are lagging, I will ask around what’s going on, but this usually has to do with specific circumstances of why certain expulsions didn’t go as expected. What matters is that I can explain the numbers. We don’t get any bonus for making more expulsions. That’s not how we work. If everyone is doing their job well, then that’s it. (Interview 10)

Emphasizing that the number of qualified expulsions did not just depend on his agents, the commander referenced the fact that other police units in Madrid occasionally arrested people who turned out to be illegalized migrants and thus brought them to the attention of the BAB. These illegalized migrants often did not meet the criteria for the category of qualified expulsions, but the BAB still had to process their cases and decide whether or not to move ahead with their deportation or not. As I was able to observe, if things were ‘going slowly’ at the BAB, agents were more open to proceeding with the deportation of non-qualified migrants who were brought to them by other police units. Some BAB agents also explained to me that they might process the deportation of migrants who were brought in by other units because they did not want to ‘offend’ or damage their good relationship with a specific agent or unit.

When I asked BAB agents about their particular interpretation of qualified expulsions, some refused to discuss the matter, while others dismissed the question – or, as one agent put it: ‘What is obvious is that we must deport the *chorizos* from Spain’ (Interview 11). The word *chorizo*, which literally means a type of pork sausage and colloquially means ‘a thief’, was used in this context by police agents as a slang term for ‘bad foreigners’.

From the answers I did receive, two issues transpired as important for agents in relation to the inclusion of police records in their implementation of qualified expulsions. First, in the most practical sense, BAB agents had direct and unmediated access to police records. A quick computer check within their own police intranet instantly provided the police record of any person in Spain. The same was not the case with regard to obtaining information on someone’s criminal or judicial record. This had to be solicited from other instances, and the police sometimes had to wait a long while before receiving an answer to their requests for such information. Second, and probably more crucially, the professionalism of the police was on the line in the question of whether police records should be included in the criteria for qualified expulsions. Several BAB agents were adamant about the merits of on-the-ground police assessment regarding the criminal involvement of individuals who were suspected and/or arrested by agents. While they had doubts about the considerations of the judiciary, they had complete confidence in the assessment of their own colleagues when it came to defining the criminality of an illegalized migrant. Here is how one BAB agent saw it:

Qualified is someone with a record. Police records translate into criminal records. If the police stop you, it’s because you committed a crime. It’s possible that sometimes the person is not charged in court, and it stays only as a police record, but the person still did it. (Interview 12)

In interviews with BAB agents, I often heard a subtle critique of the judicial system regarding its leniency in dealing with illegalized migrants. Much of the police criticism was directed at a recent trend among some judges in Spain to reject police requests to have potential deportees detained in detention facilities (Gonzalez-Beilfuss et al., 2018). Several critical reports have condemned the conditions in specially designated detention centres for foreigners (*Centros de Internamiento de Extranjeros*, hereafter CIEs) for failing to meet basic standards and safeguard the rights of detainees (Daunis, 2016; Observatorio Criminológico del Sistema Penal ante la Inmigración, 2017; Solanes Corella, 2016). In reaction to the inclination of judges not to authorize detention in CIEs for illegalized migrants who had been arrested by the police, many agents at different police units throughout Spain resorted to a new deportation technique that became infamously known as ‘express expulsions’ or ‘72-hour expulsions’. These terms refer to attempts by the police to bypass the judiciary and use their powers to detain suspects in a police station for up to 72 hours without bringing them before a judge. The police then arrest illegalized migrants in police stations and move quickly to process their deportation from the country within the 72-hour ‘window of opportunity’ they have.^10^

Lawyers and civil society actors in Spain have vehemently condemned and contested ‘express expulsions’, which often deprive migrants of their basic right to see a lawyer and to legally contest their arrest and deportation.^11^ ‘Express expulsions’ are cruely inhumane in the sense that arrested migrants have no time to prepare for their imminent deportation and are often in a state of shock about the abruptness of events: migrants are usually not given the chance to say goodbye to their loved ones, pack their belongings, claim any pending salary or debt, and so forth (Servicio Jesuita a Migrantes de España, 2015). Notwithstanding the dubious legality of this form of deportation, and the pain it inflicts on illegalized migrants, the police have adamantly defended the course of their actions, and accordingly the number of ‘express deportations’ has risen in recent years, becoming since 2013 the most prominent type of deportation in Spain (*Europa Press*, 2015).

## ‘House of cards’ and institutional racism

In the years following 2009, reports by independent NGOs, investigative journalists and critical academics (e.g. Martínez Escamilla, 2013) repeatedly claimed that the statistics concerning qualified expulsions had been manipulated by the Ministry of the Interior and deliberately mispresented to the public. For example, according to one report, in 2015 the ministry informed the public that 81% of all deportations that year were qualified expulsions, while in fact 36% of these deportations concerned people with an irregular status who might have had police records but had no criminal charges (Servicio Jesuita a Migrantes de España, 2015). This meant, the independent report insisted, that the majority of deportations in 2015 (56%) were in fact ‘non-qualified’. Arguably, as a result of these repeated contestations and embarrassing revelations by independent sources, from 2016 the Ministry of the Interior decided that it would no longer present the amount of qualified expulsions as a percentage of the total annual number of deportations (see Ministry of the Interior, 2016). Moreover, in reaction to allegations about the systematic spreading of misinformation, the ministry decided in 2017 to discontinue the publication of the annual report on the ‘Fight Against Irregular Immigration’.

Notably, for years scholars have been denouncing the fact that the majority of qualified expulsions in fact concerned illegalized migrants with no criminal records (see Brandariz-García and Fernández-Bessa, 2017; Martínez Escamilla, 2016). In 2018, a comprehensive investigation, based on figures obtained under the Spanish law of data transparency, demonstrated conclusively that since 2009 the Ministry of the Interior had been presenting false statistics about the number of qualified expulsions (Jara and Suárez, 2018). In some years, the investigation revealed, the number of qualified expulsions reported by the ministry was four times higher than the actual figure.

Misinforming the public about police actions in the field of immigration has not been uncommon in Spain in recent years. For example, in 2009 it was revealed that at some periods the Ministry of the Interior instructed police units to arrest and deport a fixed quota of illegalized migrants every month (see Barbero and Fernández-Bessa, 2013). At one point, the ministry also instructed the police to specifically focus on illegalized migrants from Morocco. While such allegations about the existence of deportation quotas and ethnic targeting were frequently voiced by independent sources, they were always denied by the ministry until 2009, when an internal classified document surfaced and the ministry had to admit to and formally refrain from these unlawful practices (*El Confidencial*, 2009). Another notorious police ill-practice concerned an intense targeting of illegalized migrants from a particular country to fill up so-called macro deportation flights. ‘Macro’ flights were organized by Frontex, by the Spanish authorities, or by both, using charter flights for the exclusive purpose of deporting illegalized migrants to specific countries. In Spain, in periods before a ‘macro’ flight was planned to depart to a certain country, the police would particularly target migrants from that country. In contrast to an insistent denial by the ministry, some BAB agents openly talked to me about trying to fill up ‘macro’ flights because ‘it’s already paid, so we should do our best to make good use of it’ (Interview 13).

My point here is not to claim that everyone who is working at Spain’s Ministry of the Interior or at the BAB is an avowed anti-immigration person (although I equally do not claim that this is not the case).^12^ My claim is that to best understand the political manoeuvres around the notion of qualified expulsions by the Ministry of the Interior, as well as the practical interpretations of the policy by police agents, we must recognize that the entire immigration field in Spain, as in many other countries, is distinctively skewed towards the demonization and exclusion of those who are politically and publicly construed as undesired Others (Kalir, 2022). In order to account for this entrenched inclination in the field, I recently coined the term ‘departheid’ to ‘somewhat intuitively capture the political will and the widespread societal acceptance of a massive investment in specifically targeting and severely sanctioning illegalized migrants’ (Kalir, 2019c: 20). Important here is to recognize that ‘departheid has both a concrete materiality – in the form of laws, fortified borders, detention centers, deportation flights, and so forth – and a spirit in the form of prevailing cultural and societal moods that inform and promote the dehumanization of illegalized migrants’ (Kalir, 2019c: 20).

Thus, by charging the police with a mission to deport ‘foreign delinquents’, the Spanish government reaffirmed and exacerbated existing practices of ethnic and racial profiling among the police. Consider here the way in which one high-ranked BAB agent explained to me the police focus on qualified expulsions:

What we are looking for is a profile of a delinquent. For example, there are countries that, given their idiosyncrasy, their culture, their lacking education, the practice of violating their women is. . . . I don’t want to mention any specific country, not to offend anyone, but . . . well . . . some countries in South America and in Central America. When it happens there, it is not considered really bad. This is very different from how we consider it here in Spain, where there is much attention to gender violence. (Interview 14)

Another BAB agent once assured me that during police raids there was a conscious attempt to minimize any disturbance to public order: ‘At a Metro exit, for example, we don’t stop those who are clearly going to or returning from work’ (Interview 15). It would be interesting to observe how the police distinguish in crowded places between illegalized migrants and all others. Importantly, in 2018, according to data gathered by the Spanish Ombudsman, people from 89 different countries were detained for the purpose of deportation. Of these, 35.5% were from Morocco, followed by 31.7% from Algeria. The next three countries in the list of most detained nationalities were also all from Africa: Guinea (5.32 %), Senegal (4.07 %) and Gambia (3.21 %). The first non-African country on the list was Colombia (1.62 %) (Defensor del Pueblo, 2019: 69–70). In other words, the vast majority of people who are subjected to arrest are non-whites from Arab countries, followed by non-whites from Latin American countries (Colombians particularly suffer from a stigmatized image among Spaniards, and many of them have a dark skin colour). The only country within the EU with a significant number of arrests is Romania (1.20 %) (Defensor del Pueblo, 2019: 69–70). Romanians also suffer from a heavy stigma in Spain, and empirical studies have shown that many of the Romanians that get arrested and deported are Roma, who are often perceived as non-white in Europe (Vrăbiescu and Kalir, 2018).

## Conclusion

Narrowing down the focus to ‘dangerous criminals’ promised to shore up the legitimacy of deportation as an appropriate sanction for a specific group of illegalized migrants. The idea that the police were now removing violent foreign criminals to protect Spanish society relegitimized deportation in the public eye and reassured the police about the importance of their work and the morality that underlay it.

In practice, however, the Ministry of the Interior left the implementation of qualified expulsions to the police, with little or no monitoring, while occasionally promoting an inflammatory discourse against illegalized migrants that framed them as a security threat. In turn, the police opted for an expansive working definition of qualified expulsions, which practically allowed them to deport whomever they suspected of being a ‘bad migrant’. The particular manner in which the police went about implementing qualified expulsions largely derived from an entrenched anti-immigration attitude that is characteristic of policymaking and policing in the immigration field in Spain, as well as in many other countries.

A dominant working assumption among many state actors in the immigration field is that undesired migrants deserve the institutional treatment they receive. The logic is circular and flippant: if migrants are illegalized, it is because they are illegal; if they are criminalized, it is because they are criminal; if they are deported, it is because they are deportable. This logic ensures that the correspondence and proportionality between an irregular civic status and the sanction of deportation are never questioned by those who implement the law. On their part, the police are simply doing their best to professionally complete their mission, as they understand and interpret it.

The dominance of the securitization of migration as a hegemonic paradigm in the management of border crossing and human mobility is evident not only in the instigation of ever more restrictive policies and the wide usage of violent and regressive measures. It is also evident – perhaps more clearly – in the implementation of policies that are purportedly progressive and accommodating for the majority of illegalized migrants. As this article has demonstrated, this is the case because the field is mired in institutionalized racism against the figure of the undesired migrant. Institutional racism is extremely effective precisely because it operates beneath the formal and often also the discursive level. Institutional racism is in action – clearly, but invisibly – in the translation of progressive policies into violent and discriminatory practices, in ways that allegedly pertain to the realm of the professionalism of the police or their commitment to their mission. In its effects on deportation and deportability, the meaning of qualified expulsions crucially depends on those who are charged with qualifying it.

We must conclude, then, that deportability becomes the key signifier of abject Others in states that govern migration first and foremost as a racialized security matter. Qualifying the deportability of migrants in more progressive ways appears to do little in terms of mitigating their vulnerability and potential for being targeted for arrest and eventual deportation by the police. A change in high-level policies regulating deportability might only be effective if a parallel systemic change is advanced on the street level of implementation. Otherwise, illegalized migrants will keep carrying their deportability as a mark of Cain, inadvertently reaffirming their culpability in the eyes of police agents who operate in an arena dominated by institutional racism and anti-immigration sentiment.

## Notes

1. I use the term ‘illegalized migrants’ to underscore the process by which states categorize and treat certain people as ‘illegal’. It includes all sorts of undocumented migrants and rejected asylum-seekers.

2. Our findings concern the functioning of the BAB and might not be representative of police practices in other parts of Spain, especially at its southern borders and in the Canary Islands.

3. For more details on the difficulties in getting and managing access to and conducting fieldwork with the Spanish National Police, see Kalir (2019b).

4. See, for example, *La Vanguardia* (2013) or Ibáñez (2012).

5. See also https://ciesno.wordpress.com/category/campana-estatal-por-el-cierre-de-los-centros-de-internamiento-de-extranjeros/ (accessed 29 January 2020).

6. See, for example, the discussion of the 2017 court decision in the case of *N.D. and N.T. vs. Spain* in Gortázar Rotaeche and Ferré Trad (2017).

7. An extensive documentary film of the events in and around the Tarajal killing is available at https://www.youtube.com/watch?v=81MQN-6IaFM (accessed 6 May 2022).

8. See, for example, Gómez Garrido (2016) for a report on the annual bulletin of SOS Racismo with respect to police discrimination against illegalized migrants.

9. See, for example, *Hoy* (2010); *ABC* (2010).

10. For more on ‘express expulsions’ and their detrimental effects on illegalized migrants, see Barbero (2018); Martínez Escamilla (2016).

11. See SOS Rasismo (2016).

12. There have been numerous occasions in recent years in which WhatsApp groups of police agents working in the migration field have been publicly exposed, showing how agents used racist and violent expressions to talk about migrants in Spain. Some agents also prided themselves on harrassing or planning to harrass migrants during their shifts. See, for example, Pinheiro and Precedo (2017).

## Interviews cited

1. Senior field officer, BEDEX, Madrid, February 2016.
2. Junior field officer, BAB, Madrid, May 2016.
3. Senior field officer, BAB, Madrid, April 2016.
4. Veteran field officer, BAB, Madrid, April 2017.
5. Junior field officer, BAB, Madrid, March 2017.
6. Senior field officer, BAB, Madrid, March 2017.
7. Commander, deportation subunit, BAB, Madrid, February 2017.
8. Junior field officer, BAB, Madrid, November 2016.
9. Commander, deportation subunit, BAB, Madrid, May 2017.
10. Commander, deportation subunit, BAB, Madrid, May 2017.
11. Junior field officer, BAB, Madrid, October 2016.
12. Junior field officer, BAB, Madrid, May 2017.
13. Junior field officer, BAB, Madrid, October 2017.
14. Junior field officer, BAB, Madrid, November 2017.
15. Junior field officer, BAB, Madrid, May 2017.

## References

[bibr1-09670106221118798] *ABC* (2010) La policía expulsa a casi 7.600 extranjeros en 2009 por cometer 24.000 delitos [The police deport around 7600 foreigners in 2009 for committing 24,000 crimes]. 24 January. Available at: https://www.abc.es/20100124/nacional-nacional/policia-expulsa-casi-extranjeros-201001241243.html (accessed 6 May 2022).

[bibr2-09670106221118798] Aja E Arango J (2006) Veinte años de inmigración en España: Perspectiva jurídica y sociológica (1985–2004) [20 Years of Immigration in Spain: Legal and Sociological Perspective (1985–2004)]. Barcelona: CIDOB.

[bibr3-09670106221118798] Amit (2020) Between the Excessive and the Effective: The Everyday Life of the Israeli Deportation Regime. Doctoral dissertation, University of Amsterdam, the Netherlands.

[bibr4-09670106221118798] Anderson B Matthew JG Paolett E (2013) The Social, Political and Historical Contours of Deportation. Berlin: Springer.

[bibr5-09670106221118798] Arenas García L García España E (2016) Identificaciones policiales y discriminación racial en España: Evaluación de un programa para su reducción [Police detections and racial discrimination in Spain: Evaluation of a program aimed at its reduction]. Boletín criminológico 22: 1–9.

[bibr6-09670106221118798] Arendt H (2018) Thinking Without a Banister: Essays in Understanding, 1953–1975. New York: Schocken.

[bibr7-09670106221118798] Armenta A (2017) Protect, Serve, and Deport: The Rise of Policing as Immigration Enforcement. Oakland, CA: University of California Press.

[bibr8-09670106221118798] Barbero I (2018) Estudio jurídico-empírico de la detención, internamiento y expulsión de extranjeros en el País Vasco: Especial examen a las expulsiones exprés [An empirical-legal study of the apprehension, detention and deportation of foreigners in the Basque Country: Special examination of express deportations]. Migraciones – Publicación del Instituto Universitario de Estudios Sobre Migraciones 45: 143–171.

[bibr9-09670106221118798] Barbero I Fernández-Bessa C (2013) Beyond surveillance: Racial profiled detention practices in everyday life. In: Webster CWR Galdon Clavell G Zurawski N Boersma K Sagvari B Backman C Leleux C (eds) Living in Surveillance Societies: The State of Surveillance. Seattle, WA: CreateSpace, 295–304.

[bibr10-09670106221118798] Batalova J Mittelstadt M (2012) Relief from deportation: Demographic profile of the DREAMers potentially eligible under the Deferred Action Policy. Washington, DC: Migration Policy Institute.

[bibr11-09670106221118798] Bauman Z (1991) Modernity and the Holocaust. Cambridge: Polity Press.

[bibr12-09670106221118798] Bosworth M Franko K Pickering S (2018) Punishment, globalization and migration control: ‘Get them the hell out of here’. Punishment & Society 20(1): 34–53.

[bibr13-09670106221118798] Bourbeau P (2011) The Securitization of Migration: A Study of Movement and Order. London: Taylor & Francis.

[bibr14-09670106221118798] Brandariz-García JA Fernández-Bessa C (2017) The managerial turn: The transformation of Spanish migration control policies since the onset of the economic crisis. The Howard Journal of Crime and Justice 56(2): 198–219.

[bibr15-09670106221118798] Cacho LM (2012) Social Death: Racialized Rightlessness and the Criminalization of the Unprotected. New York: NYU Press.

[bibr16-09670106221118798] Calavita K (2005) Immigrants at the Margins: Law, Race, and Exclusion in Southern Europe. Cambridge: Cambridge University Press.

[bibr17-09670106221118798] Calavita K Suarez-Navaz L (2003) Spanish immigration law and the construction of difference: Citizens and ‘illegals’ on Europe’s southern border. In: Perry R Maurer B (eds) Globalization Under Construction: Governmentality, Law, and Identity. Minneapolis, MN: University of Minnesota Press, 99–127.

[bibr18-09670106221118798] Campaña Estatal por el Cierre de los CIEs (2014) Paremos los vuelos: Las deportaciones de inmigrantes y el boicot a Air Europa [Stopping the Flights: The Deportation of Foreigners and the Boycott of Air Europe]. Oviedo: Cambalache.

[bibr19-09670106221118798] Daunis RA (2016) Internamiento de extranjeros en España: Análisis crítico sobre sus condiciones materiales y tratamiento legal [The detention of foreigners in Spain: Critical analysis of their material conditions and legal treatment]. In: Laurenzo Copello P Daunis Rodríguez A (eds) Colectivos en los márgenes del derecho [Collectives at the Margins of the Law]. Valencia: Tirant lo Blanch, 137–181.

[bibr20-09670106221118798] De Genova NP (2002) Migrant ‘illegality’ and deportability in everyday life. Annual Review of Anthropology 31: 419–447.

[bibr21-09670106221118798] De Genova NP Peutz N (2010) The Deportation Regime: Sovereignty, Space, and the Freedom of Movement. Durham, NC: Duke University Press.

[bibr22-09670106221118798] Defensor del Pueblo (2019) Informe anual 2018: Mecanismo Nacional de Prevención [Annual Report 2018: National Prevention Mechanism]. Madrid: Defensor del Pueblo. Available at: https://www.defensordelpueblo.es/informe-mnp/mecanismo-nacional-prevencion-la-tortura-informe-anual-2018/ (accessed 6 February 2020).

[bibr23-09670106221118798] *El Confidencial* (2009) Rubalcaba admite que la Policía fijó cupos de arrestos de ‘sin papeles’ [Rubalcaba admits the police set quotas for arresting undocumented migrants]. 16 February. Available at: https://www.elconfidencial.com/espana/2009-02-16/rubalcaba-admite-que-la-policia-fijo-cupos-de-arrestos-de-sin-papeles_209797/ (accessed 6 February 2020).

[bibr24-09670106221118798] *elDiario.es* (2014) Fernández Díaz: ‘Entre los inmigrantes irregulares se cuelan terroristas yihadistas’ [Fernández Díaz: ‘Among irregular migrants there are jihadist terrorists who sneak in’]. 6 November. Available at: https://www.eldiario.es/desalambre/fernandez-diaz-inmigrantes-irregulares-terroristas_1_4533811.html (accessed 12 April 2020).

[bibr25-09670106221118798] Ellermann A (2008) The limits of unilateral migration control: Deportation and inter-state cooperation. Government and Opposition 43(2): 168–189.

[bibr26-09670106221118798] *Europa Press* (2015) Expulsiones exprés: Inmigrantes arraigados, con engaños y sin abogado [Express deportation: Settled immigrants, being cheated and without a lawyer]. 22 April. Available at: https://www.europapress.es/epsocial/cooperacion-desarrollo/noticia-expulsiones-expres-inmigrantes-arraigados-enganos-abogado-20150422144444.html (accessed 6 February 2020).

[bibr27-09670106221118798] *Eurostat* (2018) Statistics on enforcement of immigration legislation. Available at: https://ec.europa.eu/eurostat/statistics-explained/index.php?title=Statistics_on_enforcement_of_immigration_legislation#Non-EU_citizens_ordered_to_leave_the_EU (accessed 13 January 2020).

[bibr28-09670106221118798] Fassin D (2005) Compassion and repression: The moral economy of immigration policies in France. Cultural Anthropology 20(3): 362–387.

[bibr29-09670106221118798] Feldman G (2016) ‘With my head on the pillow’: Sovereignty, ethics, and evil among undercover police investigators. Comparative Studies in Society and History 58(2): 491–518.

[bibr30-09670106221118798] Feldman G (2019) The Gray Zone: Sovereignty, Human Smuggling, and Undercover Police Investigation in Europe. Stanford, CA: Stanford University Press.

[bibr31-09670106221118798] Fekete L Webber F (2009) Foreign nationals, enemy penology and the criminal justice system. IRR European Race Bulletin 69: 2–17.

[bibr32-09670106221118798] Fernández-Bessa C Brandáriz-García JA (2016) Transformaciones de la penalidad migratoria en el contexto de la crisis económica: El giro gerencial del dispositivo de deportación [Transformation in migration-related sanctions within the context of the economic crisis: The managerial turn in the mechanism of deportation]. InDret – Revista para el análisis del Derecho 4: 1–25.

[bibr33-09670106221118798] Franck AK Vigneswaran D (2023) Hacking migration control: Repurposing and reprogramming deportability. Security Dialogue 54(6): 568–585.

[bibr34-09670106221118798] Galvin TM (2015) ‘We deport them but they keep coming back’: The normalcy of deportation in the daily life of ‘undocumented’ Zimbabwean migrant workers in Botswana. Journal of Ethnic and Migration Studies 41(4): 617–634.

[bibr35-09670106221118798] Gómez Garrido M (2016) Agresiones y racismo institucional: Una ‘costumbre’ en España [Aggressions and institutional racism: A ‘common practice’ in Spain]. La Marea, 16 September. Available at: https://www.lamarea.com/2016/09/16/agresiones-racismo-institucional-una-costumbre-espana/ (accessed 6 February 2020).

[bibr36-09670106221118798] Gonzalez-Beilfuss M Vallbe JJ Kalir B (2018) El internamiento de extranjeros: ¿Qué dicen los datos? [The detention of foreigners: What do the data say?]. Migraciones 45: 57–88.

[bibr37-09670106221118798] González-Enríquez C (2009) Spain, the cheap model: Irregularity and regularisation as immigration management policies. European Journal of Migration and Law 11(2): 139–157.

[bibr38-09670106221118798] Gortázar Rotaeche C Ferré Trad N (2017) A cold shower for Spain – hot returns from Melilla to Morocco: N.D. and N.T. v Spain ECtHR, 3 October 2017. EU Immigration and Asylum Law and Policy, 20 October. Available at: https://eumigrationlawblog.eu/a-cold-shower-for-spain-hot-returns-from-melilla-to-morocco-n-d-and-n-t-v-spain-ecthr-3-october-2017/ (accessed 29 January 2020).

[bibr39-09670106221118798] Hagan J Eschbach K Rodriguez N (2008) US deportation policy, family separation, and circular migration. International Migration Review 42(1): 64–88.

[bibr40-09670106221118798] Hall A (2012) Border Watch: Cultures of Immigration, Detention and Control. London: Pluto Press.

[bibr41-09670106221118798] Hasselberg I (2014) Whose security? The deportation of foreign-national offenders from the UK. In: Maguire M Frois C Zurawski N (eds) The Anthropology of Security: Perspectives from the Frontline of Policing, Counter-Terrorism and Border Control. London: Pluto Press, 139–157.

[bibr42-09670106221118798] Heyman J (1995) Putting power in the anthropology of bureaucracy: The Immigration and Naturalization Service at the Mexico–United States border. Current Anthropology 36(2): 261–287.

[bibr43-09670106221118798] *Hoy* (2010) La policía expulsa a casi 7.600 extranjeros en 2009 por cometer 24.000 delitos [The police deport almost 7.600 foreigners in 2009 for committing 24.000 offenses]. 24 January. Available at: https://www.hoy.es/20100124/mas-actualidad/nacional/policia-expulsa-casi-extranjeros-201001241243_amp.html (accessed 6 May 2022).

[bibr44-09670106221118798] Huysmans J (2000) The European Union and the securitization of migration. JCMS: Journal of Common Market Studies 38(5) 751–777.

[bibr45-09670106221118798] Ibáñez MJ (2012) La jueza abre una investigación por la muerte de un joven en BCN [A judge calls for an investigation into the death of a young person in Barcelona]. El Periódico, 13 January. Available at: https://www.elperiodico.com/es/sociedad/20120113/jueza-abre-investigacion-muerte-joven-1320415 (accessed 29 January 2020).

[bibr46-09670106221118798] Inda JX (2006) Targeting Migrants: Government, Technology and Ethics. Oxford: Blackwell.

[bibr47-09670106221118798] Jara Y Suárez C (2018) Los gobiernos de Zapatero y Rajoy inflaron las expulsiones de inmigrantes por delinquir [The governments of Zapatero and Rajoy inflated the number of deportations of immigrants involved in criminal activities]. El Confidencial, 11 October. Available at: https://www.elconfidencial.com/espana/2018-10-11/interior-inmigrantes-expulsiones-policia_1628395/ (accessed 6 May 2022).

[bibr48-09670106221118798] Jueces para la Democracia (2011) Conclusiones sobre migraciones y desplazamientos de personas [Conclusions concerning migrations and the displacements of people]. 10 June. Available at: http://www.juecesdemocracia.es/congresos/xxvicongreso/conclusiones/PROPUESTAS%20SOBRE%20INMIGRACION.pdf (accessed 6 February 2020).

[bibr49-09670106221118798] Kalir B (2010) Latino Migrants in the Jewish State: Undocumented Lives in Israel. Bloomington, IN: Indiana University Press.

[bibr50-09670106221118798] Kalir B (2015) The Jewish state of anxiety: Between moral obligation and fearism in the treatment of African asylum seekers in Israel. Ethnic and Migration Studies 41(4): 580–598.

[bibr51-09670106221118798] Kalir B (2017a) State desertion and ‘out-of-procedure’ asylum seekers in the Netherlands. Focaal: Journal of Global and Historical Anthropology 77(1): 63–75.

[bibr52-09670106221118798] Kalir B (2017b) Between ‘voluntary’ return programs and soft deportation: Sending vulnerable migrants in Spain back ‘home’. In: Vathi Z Kings R (eds) Return Migration and Psychosocial Wellbeing. New York: Routledge, 56–71.

[bibr53-09670106221118798] Kalir B (2019a) Repressive compassion: Deportation caseworkers furnishing an emotional comfort zone in encounters with illegalized migrants. PoLAR: Political and Legal Anthropology Review 42(1): 68–84. 10.1111/plar.12281 PMC6581017 31244521

[bibr54-09670106221118798] Kalir B (2019b) The uncomfortable truth about luck: Reflections on getting access to the Spanish state deportation field. Social Anthropology 27(S1): 84–99.

[bibr55-09670106221118798] Kalir B (2019c) Departheid: The draconian governance of illegalized migrants in Western states. Conflict and Society 5(1): 19–40.

[bibr56-09670106221118798] Kalir B (2022) On the death of Mame Mbaye: Racialism as the ultimate goal in the ‘battle against irregular immigration’. State Crime Journal 11(1): 70–89.

[bibr57-09670106221118798] Kalir B Sur M Van Schendel W (2012) Introduction: Mobile practices and regimes of permissiveness. In: Kalir B Sur M (eds) Transnational Flows and Permissive Polities. Amsterdam: Amsterdam University Press, 11–26.

[bibr58-09670106221118798] Kanstroom D (2007) Deportation Nation: Outsiders in American History. Cambridge, MA: Harvard University Press.

[bibr59-09670106221118798] Karpiak KG Garriott W (2018) The Anthropology of Police. London: Routledge.

[bibr60-09670106221118798] Kirchhoff M Lorenz D (2018) Between illegalization, toleration, and recognition: Contested asylum and deportation policies in Germany. In: Rosenberger S Stern V Merhaut N (eds) Protest Movements in Asylum and Deportation. Cham: Springer Open, 49–68.

[bibr61-09670106221118798] *La Vanguardia* (2013) El Defensor del Pueblo investiga de oficio la muerte de un interno del CIE de Barcelona [The Ombudsman investigates ex officio the death of a detainee at the CIE of Barcelona]. 4 December. Available at: https://www.lavanguardia.com/local/barcelona/20131204/54395186987/defensor-del-pueblo-investiga-muerte-cie-barcelona.html (accessed 29 January 2020).

[bibr62-09670106221118798] Lipsky M (1980) Street-Level Bureaucracy: Dilemmas of the Individual in Public Services. New York: Russell Sage Foundation.

[bibr63-09670106221118798] López-Sala A (2015) La inmigración irregular en Ceuta y Melilla en 2014: Prácticas de control y debate público [Irregular immigration in Ceuta and Melilla in 2014: Practices of control and public debate]. In: CIDOB (ed.) Anuario de la Inmigración en España 2014 [Yearbook of Immigration in Spain 2014]. Barcelona: CIDOB, 170–191. Available at: https://www.cidob.org/articulos/anuario_cidob_de_la_inmigracion/2015/la_inmigracion_irregular_en_ceuta_y_melilla_en_2014_practicas_de_control_y_debate_publico (accessed 6 May 2022).

[bibr64-09670106221118798] López-Sala A Godenau D (2016) Integrated border management and irregular migration at the South European–North African border: The case of Spain. In: Bossong R Carrapico H (eds) EU Borders and Shifting Internal Security. London: Springer, 81–100.

[bibr65-09670106221118798] Martinez VU (2004) Trabajadores invisibles: Precariedad, rotación y pobreza de la inmigración en España [Invisible Workers: Precariousness, Rotation and Poverty of Immigration in Spain]. Madrid: Libros de la Catarata.

[bibr66-09670106221118798] Martínez Escamilla M (ed.) (2013) Mujeres en el CIE: Género, inmigración e internamiento [Women in the CIE: Gender, immigration and detention]. Madrid: Editorial Gakoa.

[bibr67-09670106221118798] Martínez Escamilla M (2016) Centros de internamiento para extranjeros: Estado de la cuestión y perspectivas de futuro. Revista Electrónica de Ciencia Penal y Criminología 18-23, 1-38. Available at: https://dialnet.unirioja.es/servlet/articulo?codigo=5788852

[bibr68-09670106221118798] Menjívar C Abrego L (2012) Legal violence: Immigration law and the lives of Central American immigrants. American Journal of Sociology 117(5): 1380–1421.

[bibr69-09670106221118798] Ministry of the Interior (2010) La BEDEX de la Policía Nacional ejecuta las expulsiones de delincuentes ordenadas judicialmente [The BEDEX unit of the National Police carries out the deportations of delinquents who are indicted by the court]. 24 January. Available at: https://www.policia.es/prensa/100124_1.htm (accessed 6 February 2020).

[bibr70-09670106221118798] Ministry of the Interior (2013) Balance 2012: Lucha contra la inmigración irregular [Balance 2013: Fight against irregular immigration]. Available at: https://www.slideshare.net/ManuelBenito2/balance-2012-de-la-lucha-contra-la-inmigracin-irregular-1/11?smtNoRedir=1 (accessed 6 February 2020).

[bibr71-09670106221118798] Ministry of the Interior (2014) Balance 2014: Lucha contra la inmigración irregular [Balance 2014: Fight against irregular immigration]. Available at: http://www.interior.gob.es/documents/10180/3066430/Balance+2014+de+la+lucha+contra+la+inmigraci%C3%B3n+irregular.pdf/4a33ce71-3834-44fc-9fbf-7983ace6cec4 (accessed 6 February 2020).

[bibr72-09670106221118798] Ministry of the Interior (2015) Balance 2015: Lucha contra la inmigración irregular [Balance 2015: Fight against irregular immigration]. Available at: http://www.interior.gob.es/documents/10180/3066430/Balance+2015+de+la+lucha+contra+la+inmigraci%C3%B3n+irregular.pdf/d67e7d4b-1cb9-4b1d-94a0-9a9ca1028f3d (accessed 6 February 2020).

[bibr73-09670106221118798] Ministry of the Interior ( 2016) Balance 2016: Lucha contra la inmigración irregular [Balance 2016: Fight against irregular immigration]. Available at: http://www.interior.gob.es/documents/10180/5791067/bal_inmigracion_irregular_2016.pdf/8a040aaf-a1b6-4493-9191-cf386fd31dd4 (accessed 6 February 2020).

[bibr74-09670106221118798] Moffette D (2018) Governing Irregular Migration: Bordering Culture, Labour, and Security in Spain. Vancouver: UBC Press.

[bibr75-09670106221118798] Nyers P (2003) Abject cosmopolitanism: The politics of protection in the anti-deportation movement. Third World Quarterly 24(6): 1069–1093.

[bibr76-09670106221118798] Observatorio Criminológico del Sistema Penal ante la Inmigración (2017) Razones para el cierre de los CIE: Del reformismo a la abolición [Reasons for the Closure of the CIEs: From Reformism to Abolition]. Málaga: Universidad de Málaga.

[bibr77-09670106221118798] Pinheiro M Precedo J (2017) Loas a Hitler y amenazas a inmigrantes en chats de la policía municipal: ‘Hay que hacer cacerías contra los guarros’ [Praises to Hitler and threats to immigrants in chats of the municipal police: ‘You have to hunt down the filthy people’]. elDiario.es, 20 November. Available at: https://www.eldiario.es/politica/loas-hitler-amenazas-inmigrantes-municipal_1_3060288.html (accessed 12 April 2020).

[bibr78-09670106221118798] Servicio Jesuita a Migrantes de España (2015) CIE y expulsiones exprés: Informe annual [CIE and express deportations: Annual report]. 22 April. Available at: http://www.sjme.org/sjme/item/794-cie-y-expulsiones-expres (accessed 6 February 2020).

[bibr79-09670106221118798] Solanes Corella A (2016) Un análisis crítico de los centros de internamiento de extranjeros en España: Normativa, realidad y alternativas [A critical analysis of the detention centers for foreigners in Spain: Regulations, reality and alternatives]. Revista Telemática de Filosofía del Derecho 19: 37–76.

[bibr80-09670106221118798] SOS Rasismo (2016) Deportaciones exprés: Una vulneración de garantías mínimas [Express deportations: A violation of minimum legal protection]. 21 July. Available at: https://sosracismo.eu/deportaciones-expres/ (accessed 6 February 2020).

[bibr81-09670106221118798] Stumpf JP (2006) The crimmigration crisis: Immigrants, crime, and sovereign power. American University Law Review 56(2): 367–419.

[bibr82-09670106221118798] Schuster L (2005) A sledgehammer to crack a nut: Deportation, detention and dispersal in Europe. Social Policy & Administration 39(6): 606–621.

[bibr83-09670106221118798] Ticktin M (2006) Where ethics and politics meet: The violence of humanitarianism in France. American Ethnologist 33(1): 33–49.

[bibr84-09670106221118798] Vrăbiescu I Kalir B (2018) Care-full failure: How auxiliary assistance to poor Roma migrant women in Spain compounds marginalization. Social Identities 24(4): 520–532.

[bibr85-09670106221118798] Waxman Z (2009) Thinking against evil? Hannah Arendt, Zygmunt Bauman, and the writing of the Holocaust. History of European Ideas 35(1): 93–104.

[bibr86-09670106221118798] Weber L (2014) Deciphering deportation practices across the Global North. In: Pickering S Ham J (eds) Handbook on Crime and International Migration. Abingdon: Routledge, 155–178.

